# Creating Implicit Measure Stimulus Sets Using a Multi-Step Piloting Method

**DOI:** 10.3390/mps6030047

**Published:** 2023-05-03

**Authors:** Daniel J. Phipps, Kyra Hamilton

**Affiliations:** 1 School of Applied Psychology, Griffith University, 176 Messines Ridge Road, Mt Gravatt, QLD 4122, Australia; 2 Menzies Health Institute Queensland, Griffith University, Parklands Drive, Gold Coast, QLD 4215, Australia; 3 Faculty of Sport and Health Sciences, University of Jyväskylä, Keskussairaalantie 4, 40600 Jyväskylä, Finland; 4 Health Sciences Research Institute, University of California, Merced, 5200 N. Lake Road, Merced, CA 95343, USA

**Keywords:** implicit measures, implicit attitude, implicit association test

## Abstract

The effect of arbitrary stimulus selection is a persistent concern when employing implicit measures. The current study tests a data-driven multi-step procedure to create stimulus items using a combination of free-recall and survey data. Six sets of stimulus items were created, representing healthy food and high sugar items in children, adolescents, and adults. Selected items were highly representative of the target concepts, in frequent use, and of near equal length. Tests of the piloted items in two samples showed slightly higher implicit measure–behavior relations compared to a previously used measure, providing preliminary support for the value in empirically based stimulus selection. Further, the items reported as being the most associated with their target concepts differed notably from what one may expect from the guidelines or population consumption patterns, highlighting the importance of informed stimulus selection.

## 1. Creating Implicit Measure Stimulus Sets Using a Multi-Step Piloting Method

Over the past three decades, there has been an increasing interest in the use of experimental tasks as measures to assess non-conscious constructs such as implicit attitudes, implicit self-concepts, and approach biases. Such measures have, in general, been successful in augmenting traditional self-reported scales to improve the prediction of outcomes and have coincided with renewed interest in dual-process models to conceptualize the role of automatic processes in human behavior [1,2,3,4].

However, despite the increasing popularity and interest in dual-process research, questions remain about the validity of measures used to assess implicit constructs like implicit attitude and implicit beliefs [5,6,7,8,9]. One persistent concern raised by dual-process researchers is that the characteristics of stimulus items used may introduce extraneous variability into measures [10,11,12,13], as all currently available implicit measures require target objects and concepts to be represented by a set of stimulus items [14,15,16]. Most commonly, these stimulus sets are made up of pictures or words of prototypical exemplars that are thought to represent that category (e.g., the target concept of alcohol might be represented by names of common alcoholic beverages). Tasks then infer nonconscious constructs like implicit attitudes through patterns of responding to these stimuli in experimental tasks. The ability of researchers to create stimulus sets to represent a wide range of categories has contributed to the growing use of implicit measures in a variety of fields, including social psychology [17], health promotion [18,19,20,21], and business [22]. However, the methodology and process of stimulus selection is often treated as a minor or trivial part of the research design, as pilot testing and data-driven stimulus selection processes are rarely reported in the current dual-process literature.

This dearth of research in piloting and robust stimulus selection occurs in spite of evidence that stimulus characteristics can influence results from implicit measures. For example, several studies have produced notable changes in mean effects and correlations with outcome measures by altering stimulus in terms of context [23], modality [24], or task irrelevant stimulus-valence associations [25]. Of the potential effects of stimulus selection, one of the more subtle yet broad findings has been evidence that seemingly minor variations in the correspondence between a stimulus item and its target concept can alter the effects found on implicit measures [12,13]. This proposition is supported by experimental evidence [26], showing that, when administering multiple versions of the racial bias IAT across four experiments, IATs produced stronger effects when stimuli were rated as more representative of their target concepts. Findings such as these reaffirm the recommendation that researchers employing implicit measures should be cautious in their stimulus selection, and seek to use highly relevant stimulus items, rather than many stimuli with a range of conceptual correspondences [11].

Further, even when highly relevant stimulus items are selected, characteristics of how exemplars are used in everyday life may also affect results [27]. For example, experimental evidence has found that implicit measures are more sensitive when target stimulus comprise of words which are frequently used and familiar to the target sample [28]. While this effect is minor when all categories are equally familiar or unfamiliar to participants, the effects of stimulus familiarity and use frequency can notably change effects on implicit measures when one category has a higher use frequency than another [29].

Another common consideration in implicit measure research is the length of stimulus words in each category, as there is evidence of slower word processing when words are particularly short (three or fewer letters) or long (10 or more letters) [30]. As a result, some researchers opt to match the mean length of stimulus words between categories [29,31,32,33,34,35]. Research has demonstrated, however, that there is little evidence of variation in implicit measures as a function of word length [36]. One potential reason this effect has not been found in implicit measure studies is that, as reading skills advance, known words are generally processed as a whole, contributing to reduced variability in processing speed as a function of word length [37,38,39]. However, in younger samples where reading skills are less advanced, the same studies found significantly slower processing speeds for long compared to short words. Thus, it is a plausible hypothesis that word length should remain a consideration, but its relevance may be minimal outside of younger samples or those with a reading impairment.

### The Current Study

**Figure 1:**
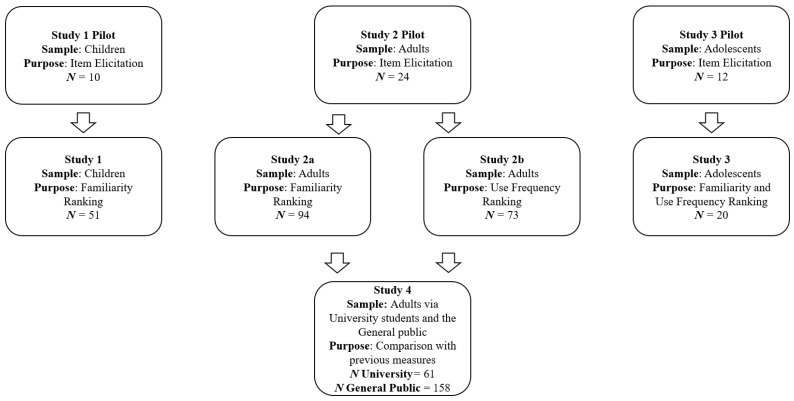
A visual summary of the current research.

Despite the potential value of good stimulus selection flagged in the current literature, research comparing stimulus sets selected based on theoretically relevant empirical data and stimulus sets selected by researchers is lacking. Thus, the current study aimed to address this research gap by outlining and testing a multi-step process to create stimulus sets for implicit measures, which accounts for several theoretically important sources of heterogeneity as a function of stimulus selection: correspondence to the construct, use frequency and familiarity, and word length. In an attempt to minimize researcher bias in selecting stimulus items, a pool of potential stimulus words will first be extracted from the target sample in a free-recall format. Then, separate samples will explicitly rate the extent to which they believe these words correspond to the target construct. Lastly, each of the words will be assessed for frequency of use and word length. Ideally, from each set of words, the researchers can select a small set of exemplars to act as stimulus items that are highly corresponding to the target concept, are at low risk of being highly unfamiliar to the target sample, and of near equal average length. In the current study, we test this process in three populations:—children, adolescents, and young adults—creating a stimulus set for the target behaviors of healthy eating and sugar intake in each population. Following piloting, we assessed the stimulus sets created for how they compared to previously employed but unpiloted measures in terms of their reliability and convergent validity. A visual summary of the research project is presented in Figure 1.

## 2. Study 1

### 2.1. Pilot

A sample of 10 children aged 6–10 years old were interviewed at a convenient location, in order to provide a pool of potential stimulus words in a free-recall format. After obtaining parental and child consent, children were asked to provide the first five things they thought of when they think of healthy foods. The children were then asked to provide the first five things they think of when they think of sugary or sweet food and drinks.

### 2.2. Participants and Procedure

A sample of 51 children aged 6–10 years old (male = 22, female = 29, *M*_age_ = 7.55, *SD*_Age_ = 1.53) were recruited to explicitly rate the extent to which they associated the words provided in the pilot with the target concepts. Participants were recruited through advertisements targeted at parents posted in parenting forums, online community noticeboards, Facebook advertisements, and an email broadcast to staff members of an Australian university.

Before beginning the survey, parents were presented with a detailed information sheet, asked to provide consent, and instructed that they may assist their child in reading and understanding, but should not attempt to influence children’s survey responses. Then, a simplified information and consent form was presented for children, which parents were asked to read and explain to their child. Once they began the survey, children were asked to rate how much they associated each of the exemplars provided in the pilot study with healthy food and sugary or sweet food and drinks (e.g., How much do apples make you think about healthy food? How much do doughnuts make you think about sweet and sugary things?). To increase readability and accessibility for children, simple language was used with a large font size, and all Likert items included a label on each possible response (0 = I do not know what that is, 1 = Not at all, 2 = A Little, 3 = Kind of, 4 = Very Much, 5 = Extremely). Researchers also recorded the length of each word in syllables and letters and its frequency of use in Australian children’s writing using the Oxford Word List [40]. Note that, compared to other corpus data, the Oxford Word List presents use frequency as a rank of sampled words. Thus, lower numbers correspond to more frequent use.

### 2.3. Results and Discussion

Study 1 aimed to create sets of potential stimulus items representing healthy eating and high sugar items in children which were rated as highly corresponding to their respective target concept, familiar to the target sample, and of near equal word length. The children’s levels of association between exemplars and healthy foods are provided in Table 1, and associations between exemplars and sweet and sugary foods and drinks are provided in Table 2.

Regarding sweet and sugary foods and drinks in the children sample, the current data suggests the category is best represented by three exemplars: candy, ice cream, and lollipop. Each of these exemplar items was rated as strongly associated with the target concept (mean correspondence ≥ 4.00), was less than three syllables and/or ten letters, had no respondents reported not knowing the word, and was in frequent use according to corpus data. It is important to note that, while the category sweet and sugary foods and drinks included both foods and drinks, the highest ranked drink was soft drink as the seventh most associated. In the healthy eating category, however, results are not as clear cut. Although nine stimulus items were rated as highly corresponding to the target concept, the length of watermelon and strawberry may cause slower reactions, and some respondents reported not knowing the word cucumber. Thus, out of caution, these words are excluded. As a result, six exemplars are chosen for the healthy eating in children category: broccoli, fruit, carrots, apples, grape, and banana.

## 3. Study 2

### 3.1. Pilot

A pool of potential stimulus words was extracted from a sample of 24 young adults at an Australian university (*M*_Age_ = 22.08, *SD*_Age_ = 7.45; 16 = female, 8 = male). This process replicated the Study 1 pilot, albeit with two changes. First, data were collected in an online survey format, rather than as an interview. Second, owing to the increased literary abilities of the young adult sample, the target concept of sweet and sugary foods and drinks was replaced with foods and drinks high in free sugar. After providing consent to participate in the study, participants were presented with a definition of free sugar. Then, participants were asked to list the first five examples which came to mind when they thought about healthy food, and then the first five examples that came to mind when they thought about foods and drinks high in free sugar.

### 3.2. Participants and Procedure

Participants for Study 2a consisted of 94 young adults aged 18 to 25 years old recruited from an Australian university (*M*_Age_ = 19.57, *SD*_Age_ = 1.99; 71 = female, 23 = male). The procedure for Study 2a mirrored that of Study 1. After completing consent and demographic information, participants were presented with the same definition of free sugar presented to the Study 2 pilot sample. Participants then rated the extent to which they associated each of the exemplars extracted from the pilot study with the concept of either foods and drinks high in free sugar or healthy foods. All exemplars were rated on a 5-point Likert scale anchored 1 = “Not at all” to 5 = “Extremely”. As the Oxford Word List used in Study 1 was designed for children only, the CORE Corpus was used as an indicator of word frequency in Study 2 [41].

After collecting Study 2a data, we concluded that word familiarity statistics extracted from Corpus data alone was likely an insufficient indicator of word use in the target sample. That is, the corpus data used is specific to the frequency of words in prose, rather than how frequently the target sample likely uses each term. As such, we recruited an additional sample of 73 young adults aged 18–25 years old from the same Australian university (*M*_Age_ = 20.17, *SD*_Age_ = 1.97, 50 female, 22 male). Participants in Study 2b were asked to rate each of the stimulus items drawn from the Study 2 pilot for how frequently they used each word (“How often do you use each of the following words?”), scored from 1 = “Never” to 6 = “Extremely often”.

### 3.3. Results and Discussion

The young adult sample’s levels of association (Study 2a) between exemplars and use frequency (Study 2b) for healthy foods and foods and drinks high in free sugar are provided in Table 3 and Table 4, respectively. As in Study 1, we sought to create a set of stimulus items which were strongly associated with the target concepts and in frequent use, conceptualized in the current study as a mean correspondence ≥4.00, and mean use frequency rating ≥2.50 or corpus frequency ≥3. In the young adult sample, 29 exemplars were rated as highly associated with the target concept of healthy food. In the interest of maintaining a small and highly relevant stimulus set [11], the 10 highest ranked suitable items were chosen: fruit, vegetables, broccoli, lettuce, bananas, oranges, spinach, salad, apples, and tomato. The exemplar vegetables was included despite being 10 characters, as evidence indicates little effect of word length in proficient readers [39]. For foods and drinks high in free sugar in the young adult sample, 16 words were found to be highly corresponding to the target construct. However, lollies, energy drinks, cordial, Sunkist, lemonade, jellybeans, maple syrup, and doughnuts were all found to be infrequently used words on both participant rating and corpus data, and were therefore excluded. The remaining stimulus items consisted of soft drink, Coke, candy, sweets, chocolate, cake, ice cream, and fast food.

## 4. Study 3

### 4.1. Pilot

A sample of 12 adolescents aged 11–14 years old (*M*_Age_ = 12.50, *SD*_Age_ = 1.17, 7 female, 5 male) were asked to provide the five words they most associated with food and drinks high in free sugar and with healthy foods in a free recall format identical to the Study 2 pilot. Participants were recruited via advertisements targeting parents on social media and using the staff email broadcast at an Australian university. After parental and adolescent consent, participants were able to access the study via a hyperlink.

### 4.2. Participants and Procedure

Participants for Study 3 were 20 adolescents aged 11–14 years old (*M*_Age_= 12.64, *SD*_Age_ = 0.90, 9 female, 11 male). Participants were recruited via advertisements targeting parents on social media and in online community groups, and using the staff email broadcast at an Australian university.

Upon clicking the advertisement, parents were presented with a detailed information sheet, and presented with a short link where their adolescent could access the study. The procedure of Study 3 mirrored that of Study 2a, with the exception that, following the rating of each item’s association with the target concepts, participants were asked to rate how frequently they used each of the stimulus words, as in Study 2b. The CORE Corpus was used as an indicator of word frequency alongside self-reported use frequency [41].

### 4.3. Results and Discussion

The adolescent samples ratings of the association between potential stimulus words and target concepts, the self-reported frequency of use, word characteristics, and corpus statistics for word use for healthy eating and foods and drinks high in free sugar are presented in Table 5 and Table 6, respectively. Mirroring Studies 1 and 2, we again aimed to create stimulus sets which were highly representative of their target concepts and in frequent use by the target sample (mean correspondence ≥4.00, and mean use frequency rating ≥2.50 or corpus frequency ≥3). Thirteen exemplars were rated as highly associated with the concept of healthy food, and of these 13 all but Brussel sprouts were in relatively frequent use. As in the young adult sample, the 10 highest ranked suitable items were selected: vegetables, carrots, salad, broccoli, apples, lettuce, banana, fruit, mango, and avocado. Of the 15 potential free sugar stimulus items extracted from the pilot sample, 10 were highly associated with the concept of free sugar. However, the word energy drink was rated as infrequently used on both rating and corpus data. The remaining nine words were: soft drink, Coke, lollies, chocolate, ice cream, cake, lemonade, and Slurpee.

## 5. Study 4

After the piloting of materials, we aimed to test the piloted measures to assess how they compared in terms of their reliability and predictive validity in comparison with previously used, unpiloted measures.

### 5.1. Participants and Procedure

Participants in Study 4 consisted of two samples: undergraduates from an Australian university, who completed measures relating to free sugar; and a community sample of Australian individuals recruited from the general public by a panel company, who completed measures relating to healthy eating. After providing informed consent, participants completed an implicit attitude measure using piloted stimuli and an implicit attitude measure using stimuli from previous research, before completing self-reported measures of behavior. In the university student free sugar sample, 67 participants completed measures; however, six participants met the implicit measure scoring exclusion criteria (e.g., excessive errors; Greenwald et al., 2003), resulting in a final sample of 61 (*M* Age = 21.54, *SD* Age = 6.24, 35 female, 25 male, 1 other). In the general population sample, 162 participants completed measures; however, four were flagged for exclusion based on the implicit measure scoring criteria [42], resulting in a final sample of 158 (*M* Age = 59.03, *SD* Age = 15.26, 83 female, 73 male, 2 other).

### 5.2. Measures

#### 5.2.1. Single Target Implicit Association Tests

Implicit attitude was assessed using the Single Target Implicit Association Test [43], administered using the IATGEN package in the Qualtrics online data collection platform [44]. The ST-IAT is a reaction time-based task used to infer participant implicit attitude towards an attitude target by comparing response times from trials when positive words share a response key with the target concept with trials where negative words share a response key with the target concept. For the ST-IATs in both samples, positive stimuli consisted of five words (good, tasty, enjoyable, nice, fun), as did negative stimuli (bad, nasty, dull, awful, boring). In the general population sample, healthy eating words for one ST-IAT were drawn from the piloting data (fruit, vegetables, broccoli, lettuce, bananas, oranges, spinach, salad), while another ST-IAT used the stimuli used in a previously published study (strawberries, rice, fruit salad, turkey filet, cucumber, apples, grapes, chicken) [45]. Similarly, in the university student population, one ST-IAT used stimuli extracted from the piloting process (soft drink, Coke, candy, sweets, chocolate, cake, ice cream, fast food), and the other used stimuli from previous research (syrup, sucrose, glucose, honey, caramel, chocolate, icing, lolly) [46,47]. All ST-IATs were scored following recommended conventions [42,43], with scores normalized for potential order effects.

#### 5.2.2. Behavior

In each sample, behavior was assessed using brief food frequency questionnaires drawn from previously validated measures of dietary consumption [48,49]. These items asked participants to respond how often they consumed common healthy or free sugar items (e.g., “Leafy green vegetables”, “Chocolate”), on an 8-point scale anchored [1] never to [8] 4+ times per day.

### 5.3. Results

In both samples, the piloted and previously used ST-IATs presented with acceptable reliability coefficients (healthy eating piloted ST-IAT α = 0.62; healthy eating previously Used ST-IAT α = 0.66; free sugar piloted ST-IAT α = 0.68; free sugar previously used ST-IAT α = 0.62), with only minor differences between ST-IATs. In the healthy eating sample, both the piloted ST-IAT (*r* = 0.28, *p* < 0.001) and the previously used ST-IAT (*r* = 0.18, *p* = 0.020) were associated with behavior, although the piloted ST-IAT had a stronger effect. In the free sugar sample, neither ST-IAT was associated with behavior, as the piloted ST-IAT had a small effect which did not meet the significance threshold (*r* = 0.09, *p* = 0.489), while the previously used ST-IAT had a negligible relationship with behavior (*r* = 0.02, *p* = 0.898).

## 6. General Discussion

The aim of the current research was to test a multi-step process to extract sets of stimulus items for implicit measures. Through this process we aimed to make informed choices on stimulus selection, accounting for the correspondence of exemplars with the target concept, the frequency of exemplar uses in common language, and the length of the exemplars. We tested this process on two prominent health behaviors: healthy eating and the consumption of products high in sugar. Testing the piloted stimulus sets in a sample of university students and a general population sample, we found little difference between piloted and previously used implicit measures in terms of the reliability coefficients, but a slightly stronger implicit measure–behavior relationship in the piloted measures.

Previous evidence has indicated variability in response times to stimulus items as a function of a stimuli’s level of correspondence to its target concept [26] and level of familiarity to its target sample [27,31]. Similarly, word length has also been theorized to effect response times [30], especially in those with less developed reading skills [37,38,39]. Thus, by using empirical data to minimize variability in stimulus items’ levels of correspondence, familiarity, and word length, the current research may represent a method of increasing the validity of research which utilizes implicit measures. Specifically, controlling for these features may serve as a pathway to increasing the accuracy of response time-based scoring metrics by reducing several potential sources of task-irrelevant variation in response times. However, in spite of evidence of the effects of stimulus characteristics on implicit measure responses, there is a dearth of research systematically investigating piloting methods and how they may affect metrics inferred from implicit measures.

In terms of the testing of implicit measures based on the extracted items, data provides preliminary support for the value in piloting stimulus sets. Although we observed the small sized implicit measure-behavior relationships typical of current dual process research [46,50,51,52], the implicit measure–behavior relationship was stronger when using the implicit measure with piloted stimulus items in both the university student and general population samples. This is consistent with previous evidence and theory that implicit measures using stimuli highly representative of their target constructs are liable to produce stronger effect sizes [11,26]. However, in contrast to our expectations, there were no systematic differences in reliability scores, as the piloted IAT had slightly higher reliability in the free sugar sample, while the previously used IAT had slightly higher reliability in the healthy eating sample. Given the fact that we expected homogenizing stimulus sets to consist of only highly relevant, familiar items to reduce the likelihood of extraneous variation in reaction times, this is somewhat surprising. However, it is also important to note that implicit measure internal consistency statistics should be interpreted with a degree of caution, given that they are subject to influences beyond the reliability of measurement, such as attitude polarity or personal importance [53]. Thus, it is difficult to assess which characteristics affected reliability in the current study, if at all. Future research may seek to address this issue using investigations in large data sets, or through improvements to the mathematical basis for estimating implicit measure reliability statistics.

Beyond evidence for the efficacy of piloted stimulus sets, the current findings also have several notable implications for broader implicit measure research. The extracted stimulus sets present a data-driven, empirically grounded representation of their target concepts in three populations. In an immediate sense, these findings have inherent value in elucidating laypeople’s understanding of free sugar and healthy food items, and thus informing stimulus selection for implicit measures focused on these key health behaviors. However, it is also important to consider that the highest rated of the extracted exemplars differ notably from published statistics and guidelines regarding dietary choices. For example, despite high sugar beverages accounting for a disproportionately high amount of sugar in children’s diets [54,55], out of the 16 exemplars provided by the child sample, the highest ranked exemplar drink, soft drink, was ranked by children as seventh, and the highest contributor to children’s sugar intake [56], juice, was ranked 14th. However, despite soft drinks making up a notably smaller portion of overall sugar intake in adults [55,56], Coke and soft drink were ranked the most associated with food and drinks high in free sugar in the adolescent and young adult samples, respectively. Similarly, with respect to healthy foods, fruits and vegetables ranked highly in all samples. While this is somewhat expected, it is important to consider that the highest rated exemplars do not match published definitions of a healthy and balanced diet [57], as grains and lean proteins were ranked as being less associated with the concept of healthy foods.

This inconsistency poses an interesting question for research design, as to whether stimulus sets should consist of those exemplars the target population rates as most corresponding with the target concept, or exemplars which cover the breadth of a concept as defined by professionals. As the aim of the current study was to test a method of piloting stimulus items which minimized any potential for extraneous input from researchers, a purely data-driven approach was employed, prioritizing the inclusion of stimulus words by their ratings of correspondence. As a result, the stimulus sets chosen may not reflect what experts define as the key exemplars for each category. An alternative approach might be to combine data with the professional opinion of experts. For example, for healthy eating exemplars in the young adult sample, the exemplars beans, fish, and eggs met the criteria of being highly corresponding to the target concept and in regular use, but were excluded as they were less highly corresponding than the selected items. Using guidelines [57] in combination with our data, a stimulus set which better covers the breadth of the concept of healthy eating in young adults might be vegetables, lettuce, fruit, broccoli, salad, eggs, tomatoes, bananas, fish, and beans to include a range of fruits, vegetables, and proteins. This is arguably a superior stimulus set than a purely data-driven approach. For example, behavior measures designed by experts to assess healthy eating often cover multiple facets of a good diet [49], rather than simply fruit and vegetable consumption. Thus, it is plausible that a stimulus set which is based on both published guidelines and empirical data may produce stronger findings through a closer correspondence to validated behavior measures that have also been designed with guidelines in mind. However, even in the current data, such a mixed approach is not always possible. In both the child and young adult samples, no grains were rated as highly associated with healthy food. Additionally, in the child sample, few proteins were provided in the free-recall experiment, and those provided were ranked as poorly associated with healthy eating in the rating task. Such a finding leaves researchers in a difficult position with regard to decisions about stimulus selection, as providing full coverage of the target concept may mean introducing extraneous variance into implicit measure results.

Such a predicament is not unique to methodology utilizing implicit measures, and the current findings are also likely to have implications for research using the more traditional self-report methods of data collection. For example, a researcher asking a simple Likert scale question on healthy eating (e.g., eating a healthy diet in the next two weeks would be [1] boring to [7] enjoyable) will receive responses based upon the participants salient definition of healthy eating. However, according to current research, this salient definition may be incongruent with conceptualizations from researchers and experts. To an extent, these findings highlight the value of providing clear definitions of key constructs in survey and self-report research. Yet, even with a clear definition provided, the extent to which findings are affected by discrepancies between provided definitions of key constructs and participants salient understanding is unclear.

### 6.1. Strengths, Limitations, and Future Directions

The current study had several notable strengths, including the use of multiple samples and an empirically grounded design. However, the current research is not without its limitations. Firstly, examining the mean level of correspondence and familiarity in samples does not account for individual differences within each sample. In the current literature, there is suggestion this could be addressed through the use of individualized stimulus items, rather than creating stimulus sets via piloting. While some studies have found this individualization technique to be useful [58,59,60], it may be impractical for many researchers in terms of creating implicit measures using currently available experimental software packages, and may in itself introduce additional variance to scores inferred from implicit measures. Future research may seek to compare the relative value of addressing stimulus variation issues through piloting or measure individualization to inform best practice implicit research. Further, while the stimulus sets created in the current study have natural value to their target populations, it is possible that cultural variations may inhibit their usefulness outside the Australian context. For example, some extracted items such as lollies or soft drink may not be familiar in US samples, where candy or soda may be more likely items. Thus, replication of the current process in alternative samples may serve as a useful avenue for future research.

### 6.2. Conclusions

The current study presented a multi-step method of creating stimulus sets for use in implicit measures including extracting potential exemplars using free-recall piloting, testing the level of correspondence between potential stimulus items and target constructs, and investigating word characteristics, such as length and use frequency, which may impact responses on implicit measures. From analysis, six sets of stimulus items were created, representing healthy eating and sugar consumption in children, adolescents, and young adults. By controlling for potentially extraneous sources of variance on reaction times, this process may represent an avenue for increasing the validity and precision of implicit measures. This was reflected in two samples where an implicit measure based upon the piloted stimuli had a slightly stronger relationship with behavior than previously used implicit measures drawn from the published literature. Thus, the current research has notable practical implications for research employing implicit measures like the implicit association test or affective misattribution procedure, providing preliminary support for the value in empirically grounded stimulus selection to ensure more accurate and valid findings, and reaffirming concerns that arbitrary stimulus selection may be an avenue of introducing biases to results. There are also practical implications for wider research design beyond the stimulus sets created, as the exemplars rated as the most corresponding to the target concepts in each sample did not accurately reflect published guidelines and known patterns of consumption. These deviations from expected patterns pose difficult questions for implicit research overall, and reaffirm the need for clear conceptualizations of key constructs for both implicit measure and traditional survey research. Future research may consider the impact of potential trade-offs when defining constructs to match the understanding of the target sample or to cover the breadth of concepts in the eyes of professionals.

## Figures and Tables

**Table 1:** Exemplar Ratings and Word Characteristics for Healthy Foods in the Child Sample.

	Sample Characteristics						Word Characteristics		
Variables	Minimum	Maximum	Mode	Median	Mean	SD	Syllables	Length	Use Frequency Rank
Broccoli	1	5	5	5	4.55	0.97	3	8	4957
Fruit	2	5	5	5	4.55	0.73	1	5	924
Carrot	1	5	5	5	4.35	0.84	2	6	1217
Watermelon	2	5	5	4	4.27	0.85	4	10	3345
Apples	2	5	5	4	4.22	0.88	2	6	856
Cucumber	0	5	5	5	4.18	1.20	3	8	4305
Strawberry	1	5	5	4	4.10	0.99	3	10	1715
Grape	1	5	5	4	4.08	0.98	1	5	4419
Banana	1	5	5	4	4.04	1.13	3	6	958
Tomato	1	5	4	4	3.73	1.20	3	6	1262
Avocado	0	5	5	4	3.69	1.46	4	7	6202
Beans	0	5	5	4	3.67	1.47	1	5	3053
Blueberry	0	5	4	4	3.65	1.35	3	9	3385
Mangos	0	5	4	4	3.61	1.25	2	6	3525
Rice	0	5	3	3	3.18	1.23	1	4	1149
Salmon	0	5	3	3	2.88	1.63	2	6	5698
Olive	0	5	3	3	2.31	1.62	2	5	Unranked

Use frequency rank refers to the ranking in the Oxford Word List, with lower numbers indicating a higher rank and more frequent use.

**Table 2:** Exemplar Ratings and Word Characteristics for Sweet and Sugary Foods and Drinks in the Child Sample.

	Sample Characteristics						Word Characteristics		
	Minimum	Maximum	Mode	Median	Mean	SD	Syllables	Letters	Corpus Frequency Rank
Candy	2	5	5	5.00	4.58	0.82	2	5	2377
Ice cream	2	5	5	4.50	4.35	0.76	2	8	416
Lollipop	1	5	5	5.00	4.33	1.08	3	8	1285
Doughnut	0	5	5	4.00	4.00	1.26	2	8	1579
Cupcakes	1	5	5	4.00	3.98	0.98	2	8	3100
Cookies	1	5	5	4.00	3.77	1.08	2	7	1117
Soft Drink	0	5	5	4.00	3.75	1.58	2	9	Unranked
Gummies	0	5	5	4.00	3.52	1.60	2	7	Unranked
Ice Block	0	5	4	4.00	3.50	1.34	3	8	5350
Coke	0	5	5	5.00	3.48	1.96	1	4	5030
Lemonade	0	5	4	3.00	3.17	1.45	3	8	1976
Strawberry Milk	0	5	4	3.00	3.08	1.57	4	14	Unranked
Chocolate Milk	0	5	3	3.00	3.06	1.45	4	13	Unranked
Juice	1	5	3	3.00	2.75	1.21	1	5	1306
Liquorice	0	5	0	1.00	2.00	1.97	3	9	3928
Gatorade	0	5	0	0.00	1.73	2.01	3	8	Unranked

Use frequency rank refers to the ranking in the Oxford Word List, with lower numbers indicating a higher rank and more frequent use.

**Table 3:** Exemplar Ratings and Word Characteristics for Healthy Foods in the Young Adult Sample.

	Familiarity (Sample 1)				Use Frequency (Sample 2)				Word Characteristics		
	Mode	Median	Mean	SD	Mode	Median	Mean	SD	Syllables	Letters	Corpus Frequency
Fruit	5	5.00	4.62	0.81	5	5.00	4.86	0.92	1	5	33.59
Vegetables	5	5.00	4.60	0.98	5	5.00	4.84	1.01	4	10	21.95
Broccoli	5	5.00	4.45	1.01	4	4.00	3.75	1.41	3	8	4.57
Lettuce	5	5.00	4.45	1.04	4	4.00	4.36	1.10	2	7	5.15
Bananas	5	5.00	4.40	0.90	4	4.00	4.01	1.45	3	7	4.34
Oranges	5	5.00	4.38	0.96	4	4.00	3.99	1.23	3	7	3.99
Spinach	5	5.00	4.38	1.12	5	4.00	3.93	1.39	2	7	5.17
Salad	5	5.00	4.35	1.00	4	4.00	4.33	1.34	2	5	19.74
Apples	5	5.00	4.34	0.99	4	4.00	4.15	1.11	2	6	11.32
Grapes	5	5.00	4.34	0.98	4	4.00	3.89	1.28	2	6	5.75
Kale	5	5.00	4.34	1.27	1	2.00	2.81	1.58	1	4	2.53
Tomatoes	5	5.00	4.26	1.08	5	5.00	4.30	1.42	3	8	15.02
Avocado	5	5.00	4.24	1.13	5	4.00	4.11	1.33	4	7	2.46
Zucchini	5	5.00	4.24	1.22	4	3.00	3.42	1.52	3	8	2.52
Peas	5	5.00	4.23	1.11	2	4.00	3.62	1.52	1	4	6.06
Lentils	5	5.00	4.22	1.21	1	2.00	2.75	1.54	2	7	1.28
Strawberries	5	4.00	4.22	0.92	5	5.00	4.37	1.26	3	12	3.68
Fish	5	5.00	4.19	1.15	4	4.00	3.77	1.55	1	4	83.08
Legumes	5	5.00	4.18	1.26	2	2.00	2.62	1.48	3	7	1.06
Cabbage	5	5.00	4.16	1.20	2	3.00	3.04	1.37	2	7	4.50
Beans	5	5.00	4.15	1.19	4	4.00	3.66	1.48	1	5	18.82
Corn	5	4.50	4.14	1.10	4	4.00	3.60	1.34	1	4	26.62
Blueberries	5	5.00	4.13	1.14	4	4.00	3.77	1.43	3	11	2.00
Chickpeas	5	5.00	4.10	1.24	2	3.00	3.04	1.67	2	9	0.83
Nuts	5	4.00	4.05	1.09	4	4.00	3.64	1.37	1	4	21.31
Chia	5	5.00	4.04	1.26	2	2.00	2.81	1.54	1	4	0.50
Quinoa	5	4.50	4.03	1.27	1	2.00	2.51	1.50	2	6	1.00
Almonds	5	4.00	4.00	1.21	4	3.00	3.27	1.45	2	7	2.89
Eggs	5	4.00	4.00	1.14	6	4.00	4.29	1.53	1	4	32.53
Whole Grains	5	4.00	3.98	1.15	4	4.00	3.30	1.48	3	11	0.43
Grains	5	4.00	3.97	1.11	4	4.00	3.52	1.42	1	6	6.72
Oats	4	4.00	3.97	1.09	3	4.00	3.77	1.45	1	4	2.31
Chicken	4	4.00	3.84	1.14	5	5.00	4.55	1.41	2	7	47.47
Olives	5	4.00	3.80	1.28	2	3.00	3.07	1.58	3	6	3.64
Tofu	5	4.00	3.77	1.26	1	2.00	2.99	1.74	2	4	3.18
Meat	5	4.00	3.68	1.26	5	5.00	4.48	1.41	1	4	43.24
Wheat	5	4.00	3.60	1.19	4	4.00	3.32	1.46	1	5	11.97
Red Meat	4	4.00	3.57	1.27	5	4.00	3.99	1.51	2	7	1.62
Yogurt	3	3.00	3.47	1.15	4	4.00	3.81	1.40	2	6	6.57
Trail mix	3	3.50	3.46	1.16	2	2.00	2.33	1.30	2	8	0.21
Juice	3	3.00	3.20	1.06	2	4.00	3.58	1.42	1	5	26.55
Bread	3	3.00	3.16	1.23	5	5.00	4.58	1.09	1	5	33.49
Pasta	3	3.00	3.14	1.09	5	5.00	4.47	1.21	2	5	10.32
Cheese	2	3.00	2.93	1.23	5	5.00	4.34	1.31	1	6	36.26

Note. Use frequency refers to uses per million words in the CORE Corpus. Higher use frequency indicates more frequent use.

**Table 4:** Exemplar Ratings and Word Characteristics for Foods and Drinks High in Free Sugar in the Young Adult Samples.

	Association (Sample 1)				Use Frequency (Sample 2)				Word Characteristics (Data)		
	Mode	Median	Mean	SD	Mode	Median	Mean	SD	Syllables	Letters	Corpus Frequency
Soft Drink	5	5.00	4.57	0.91	3	3.00	2.51	1.34	2	9	6.89
Lollies	5	5.00	4.56	0.91	2	2.00	2.25	1.23	2	7	0.58
Coke	5	5.00	4.48	1.13	3	3.00	2.48	1.47	1	4	37.51
Candy	5	5.00	4.46	1.04	1	1.00	1.59	1.34	2	5	11.45
Energy Drinks	5	5.00	4.43	1.16	1	1.00	1.53	1.43	4	12	0.36
Sunkist	5	5.00	4.27	1.18	1	1.00	1.38	1.25	2	7	0.02
Cordial	5	5.00	4.26	1.24	1	1.00	1.33	1.25	2	7	1.32
Sweets	5	5.00	4.26	1.01	1	2.00	1.97	1.30	1	6	3.70
Chocolate	5	4.00	4.21	0.83	4	3.00	3.07	1.08	3	9	27.13
Lemonade	5	5.00	4.21	1.17	2	2.00	1.96	1.15	3	8	2.60
Jellybeans	5	5.00	4.13	1.20	1	1.00	1.18	1.11	3	10	0.08
Cake	5	4.00	4.11	1.04	2	2.00	2.07	1.11	1	4	26.02
Maple Syrup	5	4.00	4.06	1.12	1	1.00	1.47	1.12	4	11	0.77
Ice Cream	5	4.00	4.05	1.03	3	3.00	2.70	1.04	3	9	10.17
Doughnuts	5	4.00	4.03	1.16	1	2.00	2.08	1.23	2	9	1.32
Fast Food	5	4.00	4.01	1.06	1	3.00	2.59	1.37	2	9	4.57
McDonalds	5	4.00	3.86	1.20	4	3.00	2.95	1.41	2	9	2.45
Flavoured Milk	4	4.00	3.74	1.19	0	1.00	1.16	1.11	4	14	0.04
Juice	4	4.00	3.73	0.95	3	3.00	2.60	1.29	1	5	16.79
Cookies	4	4.00	3.70	1.11	3	3.00	2.67	1.04	2	7	7.28
Muffins	4	4.00	3.59	1.19	2	2.00	1.96	1.32	2	7	1.64
Pastries	4	4.00	3.52	1.23	1	1.00	1.63	1.12	2	8	1.30
Biscuits	4	4.00	3.51	1.17	2	2.00	2.30	1.16	2	8	4.38
Sugar in Coffee	3	3.00	3.45	1.21	0	2.00	1.99	1.59	5	15	0.08
Muesli Bars	3	3.00	3.13	1.26	1	1.00	1.63	1.37	3	11	0.04
Pie	3	3.00	3.09	1.22	1	1.00	1.59	1.29	1	3	15.43

Note. Corpus frequency refers to uses per million words in the CORE Corpus. Higher Corpus frequency indicates more frequent use according to corpus data.

**Table 5:** Exemplar Ratings and Word Characteristics for Healthy Foods in the Adolescent Sample.

	Associations				Use Frequency				Word Characteristics (Data)		
	Mode	Median	Mean	SD	Mode	Median	Mean	SD	Syllables	Letters	Corpus Frequency
Vegetables	5	5.00	4.50	1.00	3	4.00	3.89	0.99	4	10	21.95
Carrots	5	5.00	4.50	0.61	3	4.00	3.74	0.81	2	7	6.26
Salad	5	5.00	4.50	0.69	4	4.00	3.68	1.06	2	5	19.74
Broccoli	5	5.00	4.35	1.23	3	3.00	3.42	1.26	3	8	4.57
Apple	5	4.50	4.30	0.86	3	3.00	3.35	0.99	2	6	11.32
Lettuce	5	5.00	4.30	1.08	3	3.50	3.61	0.85	2	7	5.15
Bananas	5	4.50	4.25	0.85	4	4.00	3.55	0.83	3	7	4.34
Fruit	5	4.00	4.20	0.89	4	4.00	3.80	0.95	1	5	33.59
Mango	4	4.00	4.20	0.83	3	3.00	3.60	0.82	2	5	2.62
Avocado	5	5.00	4.20	1.28	3	4.00	3.74	0.99	4	7	2.46
Strawberry	5	4.00	4.10	1.07	3	4.00	3.74	0.93	3	12	3.68
Brussel Sprouts	5	5.00	4.10	1.29	2	2.00	2.40	1.06	3	14	0.08
Oranges	5	4.00	4.10	1.07	3	3.00	3.32	0.82	3	7	3.99
Chicken	4	4.00	3.95	0.69	4	4.00	4.00	0.79	2	7	47.47
Yoghurt	4	4.00	3.95	0.76	4	4.00	3.65	0.88	2	6	6.57
Cauliflower	5	5.00	3.95	1.43	2	2.50	2.75	0.86	4	11	1.74
Spinach	5	5.00	3.90	1.65	3	3.00	3.31	0.95	2	7	5.17
Nuts	4	4.00	3.75	1.02	3	3.00	3.39	1.04	1	4	21.31
Rice	3	3.00	3.50	0.95	3	3.00	3.53	1.07	1	4	27.42
Bread	3	3.00	3.35	0.99	4	4.00	4.15	0.75	1	5	33.49
Weetbix	2	3.00	3.35	1.14	2	3.00	2.88	1.22	2	7	0.09
Smoothies	3	3.00	3.25	0.97	3	3.00	3.40	1.10	2	9	1.09

Note. Corpus frequency refers to uses per million words in the CORE Corpus. Higher Corpus frequency indicates more frequent use according to corpus data.

**Table 6:** Exemplar Ratings and Word Characteristics for Foods and Drinks High in Free Sugar in the Adolescent Sample.

	Associations				Use Frequency				Word Characteristics (Data)		
	Mode	Median	Mean	SD	Mode	Median	Mean	SD	Syllables	Letters	Corpus Frequency
Coke	5	5.00	4.60	1.10	2	3.00	2.88	1.32	1	4	37.51
Brownies	5	5.00	4.50	0.95	2	3.00	3.06	1.11	2	8	1.32
Soft drink	5	5.00	4.45	1.10	3	3.00	3.37	0.96	2	9	6.89
Lollies	5	5.00	4.45	1.05	3	3.00	3.45	0.94	2	7	0.58
Chocolate	5	5.00	4.45	0.69	4	4.00	3.75	1.07	3	9	27.13
Ice Cream	5	5.00	4.40	0.82	5	4.00	3.85	1.04	3	9	10.17
Cake	5	5.00	4.30	0.98	3	3.00	3.21	0.92	1	4	26.02
Lemonade	5	5.00	4.25	1.21	3	3.00	3.32	0.95	3	8	2.60
Energy Drinks	5	5.00	4.25	1.07	2	2.00	2.20	1.01	4	12	0.36
Slurpees	5	5.00	4.15	1.46	2	3.00	3.00	1.20	2	8	0.34
Jelly	4	4.00	3.95	1.00	2	2.00	2.47	0.90	2	5	5.81
Chips	4	4.00	3.65	1.35	4	4.00	3.63	0.90	1	5	16.00
Cordial	5	3.50	3.55	1.36	2	2.00	2.44	0.96	2	7	1.32
Biscuits	3	3.00	3.45	0.83	3	3.00	2.89	0.81	2	8	4.38
Juice	4	3.50	3.40	1.19	3	3.50	3.55	1.10	1	5	16.79

Note. Use frequency refers to uses per million words in the CORE Corpus. Higher use frequency indicates more frequent use.

## Data Availability

The data presented in this study are openly available on the Open Science Framework at https://doi.org/10.17605/OSF.IO/4ZDWJ, reference number 4ZDWJ.
